# The Clinical Nurse Specialist as the Manager of the Family Medicine Clinic: A Hybrid Solution Between Four Major Commonwealth Realms

**DOI:** 10.3390/healthcare13050524

**Published:** 2025-02-27

**Authors:** Dawid Karczewski, Jennifer M. L. Stephens, Tomasz Karczewski

**Affiliations:** 1 Cranston Ridge Medical Clinic, Calgary, AB T3M 3A9, Canada; 2 Fay W. Whitney School of Nursing, University of Wyoming, Laramie, WY 82071, USA

**Keywords:** nursing leadership, advanced practice nurse, humanist nursing theory, Clinical Nurse Specialist (CNS), family medicine, nurse-led clinic management, CRMC model, healthcare leadership, flat organizational structure, RN prescriber

## Abstract

**Background/Objectives:** There exist several interconnected issues that hinder the development of family medicine in Commonwealth realms such as the United Kingdom, Canada, New Zealand, and Australia. These issues affect both the medical and nursing professions. Family physicians, in most countries including the United Kingdom, are not considered “specialists” and are called “general practitioners” instead. The term GP is an outdated and potentially demeaning term relegated to the early 20th century when they did not receive any more than a few rotations as staff grades before being allowed to run their own community clinic. Registered nurses often cover a minor and subaltern role when working as practice nurses in the UK. They are often replaced by cost-effective, licensed practical nurses in most other English-speaking countries. Nurse practitioners in the UK, though being de facto entirely equal to family physicians in countries like Canada, Australia, New Zealand, and most US states, do not hold a special registration status. Their training is not defined in specific legislation, and they do not function as alternatives to general practitioners in the sense that patient may register with them alone which is often the case in Canada or the USA. Family medicine is, therefore, generally left lacking leadership with members of the medical and nursing professions often struggling for “power” in a way that undermines the foundation of family medicine which is to serve the health needs of a large population ranging from children to seniors. **Methods:** The purpose of this paper is to describe a solution to management in a Family Medicine Clinic in Calgary, Alberta, Canada. **Results:** In this model, the physician-nurse team developed a highly trained role of Clinical Nurse Specialist which serves as an overall clinic manager. **Conclusions:** The implementation of the Clinical Nurse Specialist-led model in family medicine has improved clinic efficiency, patient access, and provider satisfaction. This approach demonstrates a viable framework for enhancing primary care management across Commonwealth realms.

## 1. Introduction

Historically, throughout most of the Anglosphere, family physicians or “general practitioners” in the UK and Australia have run small medical businesses [1]. The hierarchy of their structure has always been set with the physician at the top and all other professionals below them to serve them. Governments have concentrated their efforts and investments in hospital and specialist care, almost completely overlooking the primary care sector. This was left to the “general practitioners” [2]. With the turning of the twenty-first century, many changes happened in society. Family physicians completed a full-fledged speciality in order to attain their titles, registered nurses were expected to hold a bachelor’s degree to practice nursing, and postgraduate nursing education was required to work in primary care due to the fact that nurses often find themselves alone with the patient, having to make crucial decisions on their own. Due to this phenomenon, in the Commonwealth realms and the USA, advanced practice nursing (APN) was born and developed into two main branches that gave birth to two new professional profiles: the nurse practitioner (NP, advanced nurse practitioner in the UK) and the Clinical Nurse Specialist or CNS [3]. The former initially appeared in Colorado with Dr. Loretta Ford [4], subsequently in Canada, and then was introduced in the UK and the rest of the Commonwealth realms to relieve the burden of family physicians and increase access to healthcare.

The new century also resulted in other sociocultural changes to society which resulted in the general public becoming more aware of health issues [5]. Needs increased in parallel with the increase in population, so demands followed and are still on the rise. Government regulations increased the complexity of care delivery in clinical settings, and as a result, threats to public health have been snowballing. The stress level that encumbers family physicians has reached unprecedented levels [6,7,8,9]. Family physicians and family nurse practitioners both in the Commonwealth realms and in the USA cannot possibly manage their own panels and clinics contemporarily. It is impractical, dangerous to patients, and unhealthy for the professionals working in these facilities. The number of tasks assigned to medical office assistants in Canada, medical assistants in the USA, and even medical secretaries in the rest of the Commonwealth realms is increasing without adequate professional training being offered to them [10]. The pyramidal structure with the physician at the top and all other professionals below to serve them is collapsing under its own unstable and unsustainable pressure. This model no longer fits the reality of today’s healthcare environment and demands. A new model is needed a linear model, a “flat organization structure” model tested in Calgary, Canada, has proven to be more beneficial, practical, and healthier for both patients and members of the team. This model is called the “Cranston Ridge Medical Clinic model” or “CRMC model”. The British have unknowingly laid the grounds for this model over the past few decades since the introduction of the profile of the practice nurse. As mentioned, the CRMC model needs to be better adopted in the rest of the Commonwealth realms, especially in North America. However, North America shows way more flexibility in recognizing the advanced status of certain registered nurses depending on their postgraduate degrees and years of experience. For example, Canada recognizes RNs with a master’s degree and at least three years of practice in a specialist environment as “Clinical Nurse Specialists” or CNSs [11]. These are considered Advanced Practice Nurses or ANPs at the same level as the Family Nurse Practitioners, even though their roles and scopes of practice differ [3,12]. The practice nurse is considered band six in the UK, while advanced nurse practitioners are band seven [13]. Those practice nurses who have completed postgraduate degrees specific to general practice may also have an annotation of specialist practice for general practice by the Nursing and Midwifery Council [14]. Therefore, across the Commonwealth realms, there are similitudes and parallels in how Clinical Nurse Specialists are recognized and regulated. This makes the proposed new model relevant across all the largest jurisdictions among the current 15 realms. This study focuses on Canada, New Zealand, Australia, and the United Kingdom because these are the major Anglosphere countries that legally recognize RN prescribers. While each jurisdiction has unique regulatory structures, they share a foundational framework for nurse prescribing, making the CRMC model particularly relevant.

## 2. Materials and Methods

The notion that only physicians can run the family medicine clinical environment is outdated. It mirrors the idea that only the physician is (the most) knowledgeable in the healthcare setting when this is no longer the case. A few decades ago, this physician-hierarchy model could have been justified because nurses hardly had a university degree; they were trained at the very local level, directly in hospitals, at the bedside. Nurses rarely had any academic-level education, and their training was primarily practical and included a limited knowledge of anatomy, pathophysiology, pharmacology, immunology, epidemiology, and management. This is no longer the case today. Nurses, like all other health professionals, including physicians, study extensively at the undergraduate level and have the opportunity to deepen their knowledge at the postgraduate level through master’s and doctoral degrees. In some countries, like the United States, the United Kingdom and, recently, in Canada, nurses have had the possibility to complete both philosophy and professional doctorates. This allows the nursing profession to boast some of the most educated professionals among its members, knowledgeable in clinical subjects, nursing theory, and managerial skills.

Data from the CRMC pilot implementation demonstrate clear benefits. Since the introduction of RN Prescribers, patient wait times have dropped from an estimated average of 7 days to same-day access. Additionally, early data suggest that the intervention has reduced unnecessary ER visits and optimized physician workload. The methodology employed for the CRMC model by the pilot project site, the Cranston Ridge Medical Clinic (CRMC) is based on the nursing theory of Roy of humanism and the principle of “veritivity” [15] as well as on the philosophy of Rogers that introduces the idea that nursing science being used creatively for the betterment of human beings, thus advancing health and well-being for all [16,17,18]. However, the model proposed and adopted by CRMC envisages that changes may be applied to it when adopted by other clinics in other communities and jurisdictions. Therefore, it is flexible, and it is in line with Rogers, who postulated that change is inevitable and that nurses can accelerate it by offering new norms through creative transformation and pandimentional awareness, which in turn promotes individualized care as opposed to the less valuable standardized one [16,17,18]. This study evaluates the impact of RN Prescribers on accessibility, cost-efficiency, and physician workload reduction. A structured evaluation method was implemented, including pre-/post-intervention comparisons, trend analysis, and qualitative patient feedback.

### 2.1. Implementation of the CRMC Model

The CRMC model was developed in response to a critical need for innovative, cost-effective healthcare solutions in the Commonwealth realms. Due to the global shortage of primary care providers, this model is already attracting interest from clinics in both Canada and New Zealand. Early-stage discussions are underway to explore its feasibility for broader adoption. Additionally, the South Calgary Primary Care Network (PCN) is working in collaboration with CRMC to evaluate whether this model could be expanded across the province and potentially presented to the Government of Alberta for wider implementation. The CRMC model tested by Cranston Ridge Medical Clinic in Calgary, Canada, was a hybrid that draws from both the British practice nurse and specialist nurse for general practice and the Canadian Clinical Nurse Specialist. It created the profile of the Clinical Nurse Specialist for Family Medicine, which, though innovative for Canada, already has a counterpart in the UK, New Zealand, and Australia. However, this model did not simply copy that of the three other major Commonwealth realms. It expanded its scope to address the needs of the local community and clinic settings.

**Figure 1:**
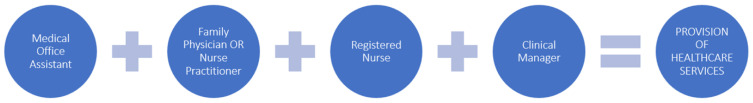
CRMC’s linear organization structure.

Thanks to their postgraduate education and extensive clinical practice, Clinical Nurse Specialists possess advanced clinical, research, and managerial skills. This, however, cannot be stated for medical school or specialist program graduates, where managerial skills are hardly taught anywhere in favour of more in-depth pre-clinical and clinical subjects, respectively. In addition, registered nurses who complete specific advanced training may be eligible to prescribe in three out of the major realms: the UK [19], Canada [20,21], and New Zealand [22,23], with Australia currently considering similar legislation [24]. Based on this, the staff at CRMC created a professional profile that concentrates the roles of practice manager, clinical support, quality controller, and educator in the CNS. This APN position, though demanding, benefits patients who may access services for urgent appointments for minor acute ailments without having to wait for days or weeks on end and the primary care providers, both physicians and nurse practitioners, who see a significant reduction in overbookings. It also benefits the staff members who can be trained in-house for the clinic’s specific tasks, from basic medical assistantship to more advanced clinical skills like early warning scores and basic life support training. According to the model developed by CRMC, the CNS is also responsible for ensuring that physicians, nurses, and even medical office assistants maintain their currency of practice and up-to-date knowledge scheduling and, at times, organize continuing professional development courses tailored to their individual clinical needs. The clinic managerial profile is integrated into a flat organization structure that is, in fact, linear (Figure 1).

In this structure, all team members in the clinic are equally needed, respected, and appreciated by the rest. They all participate within their capacity and scope’s limits to the best delivery of healthcare services to patients, and their effort is equally recognized. This structure completely abolishes the pyramidal organization that has been adopted until now. Though it was initially received with suspicion and sometimes resistance by physicians and nurse practitioners, it has eventually been appreciated by all members of the team for reducing costs, the need to outsource services, the need to hire multiple staff members to complete the tasks, and reducing waiting times for patients, which, in turn, reduced the number of complaints. While initial resistance from physicians and nurse practitioners was noted, strategic role integration and workflow adjustments have significantly mitigated these challenges. Lessons learned from the CRMC model include the importance of structured transition plans, stakeholder engagement, and financial incentive alignment. Adopting a Clinical Nurse Specialist for family medicine based on this model has been successful for many reasons.

### 2.2. The CNS as the Practice Manager

Taking advantage of the health economics courses taught at the level of a master’s degree, the CNS in the CRMC model is responsible for formulating and regularly updating the business plan, including keeping track of incomes and expenses. The Registered Nurse would be responsible for providing medical equipment orders and deliveries to the clinic, a task that could also be shared with senior medical office assistants. Functioning also as manager for the provision of services, the CNS of CRMC is responsible for the structuring of the hiring process and the adoption of good and fair hiring standards that promote good service provision by utilizing a standardized process that puts an end to parochial behaviours that often plague family medicine everywhere. Instead, they should hire only those who deserve it based on their merits. Finally, the CNS is responsible for liaising with patients and staff members when concerns or complaints are raised and for designing a schedule that matches the needs of the clinic with the staff’s individual skills.

### 2.3. The CNS as a Clinical Support

The possibility of RNs expanding their scope of practice in significant parts of the Commonwealth realms comes with the advantage of offering faster access to patients with minor ailments who cannot wait for days on end to see their physician or nurse practitioner but still do not meet the threshold to attend the emergency department. This, in turn, diminishes the burden on primary care providers and allows them to concentrate more of their time on more complicated patients with chronic conditions. In addition, this role offers an excellent opportunity to those who wish to gain some more knowledge and insight into what it feels like to independently consult patients, including roles and responsibilities, in a family medicine setting before applying for family nurse practitioner training. Regarding this last point, it would be necessary to state that, in New Zealand, RN prescribers in the primary care setting undergo a 320 h prescribing practice as part of their training before being allowed to prescribe on their own [22]. In Canada, such requirements are not part of how the profession is regulated. However, the method adopted by CRMC to overcome this difference and assure safe standards of practice for RN prescribers in the family medicine setting will be further analysed under the educator section below.

### 2.4. The CNS as a Quality Controller

Standardized training is already in place through the RN Prescriber Internship for Family Medicine, developed in collaboration with the College of Registered Nurses of Alberta (CRNA) and Athabasca University. This structured program ensures RN Prescribers are well-equipped with clinical, prescribing, and practice management skills. One of the fundamental requirements for registered nurses to be considered specialists in all Commonwealth realms is possessing a postgraduate degree, a master’s or a doctorate. This level of education assures that the nurse is versed in research quality control and quality improvement projects. Nurses with advanced education know how to conduct research and analyse relevant data. In addition, they also know how and where to obtain high-standard and clinically valid, reliable information that may inform their practice and the choices they make in writing clinical protocols for local use. CNSs ensure that the clinical environment is safe, infection risks are kept to a minimum, and the working environment complies with labour codes through regular audits and feedback provision to the staff. The CNS would also make sure that patients are prioritized correctly according to a traffic light system developed in collaboration with the primary care providers to streamline patients and assign them to the correct service and provider based on the urgency of their needs. CRMC developed a system allowing red and yellow code patients to be seen by the RN prescriber on the same day for acute minor ailments if a slot with the primary care provider is unavailable. In contrast, green code patients would then be able to be seen within seven calendar days by their physician or nurse practitioner.

### 2.5. The CNS as an Educator

The CNS, possessing at least a master’s degree, is well-positioned to offer or facilitate educational opportunities for current and aspiring staff members in a clinic. On the basis of this model and driven by the idea of improving health delivery and life opportunities through education, CRMC has developed several programs for registered nurses and medical office assistants.

The two RN prescriber internships, one of six and the other of 12 months’ duration were the initial programs set up by the CNS with the help of the College of Registered Nurses of Alberta. These were specifically designed to equip RN prescribers with the necessary skills and knowledge that will guarantee the safety of practice, a solid and robust basis on which to build one’s own practical advanced nursing experience, and uniformity of methods, including strict adherence to the legislation regulating them in Alberta [25]. The internships prepare registered nurses with a postgraduate education who wish to become CNS in the family medicine setting to complete 1197 and 1950 h, respectively. Both options include the completion of several courses in addition to the one necessary to become a nurse prescriber in the province, as well as hands-on practice. These programs will slowly prepare nurses to be able to safely utilize tools to formulate guided diagnosis, prescribe appropriate medications, request needed tests and investigations, and interpret their results. Though it would be an honour for CRMC to inspire more and more clinics to adopt this model, to date, no other location in the Commonwealth offers such comprehensive and structured training to RN prescribers in primary care.

A further example of programs available to registered nurses at CRMC that are run and administered by the CNS is the Canadian Experience Program for the Internationally Educated Nurse, which is aimed at newly immigrated RNs who recently obtained their registration and struggle to find a position in Alberta due to their lack of Canadian experience. This program also allows nurses who hold only a diploma in nursing to use CRMC as a base to complete their RN-to-BScN through the University of Athabasca, the post-secondary institution to which CRMC is affiliated.

The CNS, however, runs professional development programs for all other staff members, including physicians, nurse practitioners, and medical office assistants; among these, the CNS provides BLS training through accreditation by the Heart and Stroke (a sister foundation to the American Heart Association in Canada), infection control courses and updates, results communication training for MOAs, and teaching of the Modified Early Warning Scores of MEWS, which only occurred in the last 12 months, since their systematic introduction to all patients coming through the doors of the clinic saved the lives of more than 40 patients with subclinical myocardial infarction.

Finally, again designed by the CNS and administered in partnership with a clinical radiology firm in Calgary, CRMC has developed a medical office assistant diploma program that offers infinitely better preparation for MOAs tailored to the needs of the employer and the local community than those offered by local colleges that instead charge students exorbitant and often inaccessible tuition fees.

## 3. Results

The model developed by CRMC has led to several advantages. The introduction of the concept of urgent appointments that do not compromise the schedule and access to services for non-urgent patients has de facto transferred the provision of same-day appointments from the physician and the nurse practitioner to the clinical specialist nurse with prescribing rights. In turn, this has generated the necessity to create a standardized traffic light system developed in cooperation with physicians, nurse practitioners, registered nurses (CNS), and senior medical office assistants. This tool was specifically developed to be easy for medical office assistants at the reception as it would match the codes they used to book appointments on the electronic medical record. The pathways utilized by the CNS prescriber were also developed to match those codes and streamline the triaging of patients. This entire process has greatly reduced waiting times for patients to access services for urgent and non-urgent appointments.

By applying this model, CRMC has dramatically improved the knowledge, skills, and qualifications of all clinic team members, from MOAs to PCPs. MOAs were trained to utilize a tool that would allow them to discern what condition is to be considered urgent and which one is more chronic. The Clinical Nurse Specialists expanded their scope of practice to meet the needs of patients with urgent requests; physicians and nurse practitioners could spend more time concentrating on complex patients and gain more experience in those conditions. The model, therefore, has resulted in better and more effective care.

The capacity of one Clinical Nurse Specialist to cover multiple roles and aspects of care provision, management, education, and research has resulted in reduced expenses for the clinic’s budget. The South Calgary Primary Care Network (SCPCN) is working with CRMC to assess the financial feasibility of expanding this model across Alberta. Current funding discussions propose covering RN Prescribers at CAD 91,260 per year, which is 73% more cost-effective for the government than paying locum physicians. This funding approach aims to reduce costs while maintaining patient access to urgent and semi-urgent care. At the same time, the CRMC model achieved increased satisfaction levels among all team members. The most significant contributor reported that the cause for this was the implementation of the flat organization structure, which allowed for and promoted the recognition of professional roles and achievements of all staff members without favouring one over the other. This eventually resulted in increased motivation where all could say that they mattered, resulting in greater retention of team members and an end to the eternal revolving door for staff. Key improvements following RN Prescriber integration at CRMC include same-day access for urgent conditions, reducing prior wait times of 4–10 days, reduction in ER visits due to RN-led early interventions, and more physician availability for complex case management. In brief, the introduction of the CNS-led model in CRMC improved access to service, patient satisfaction, finances, education, staffing levels, and resource efficiency, including human resources.

## 4. Discussion

The CRMC model was initially met with considerable resistance and scepticism by physicians and nurse practitioners. They felt threatened by the perception of loss of power, which eventually they realized they never had to begin with, and that power was always with the patient, as it should be in patient-centred healthcare [26]. The patient should be placed at the centre with healthcare professionals, physicians, and nurses included, rotating around them, all fulfilling their roles and duties to the highest level for their individual licensure. Registered nurses were also afraid of taking up so much responsibility, covering multiple roles. Nonetheless, the CRMC model allowed nurses to develop and expand their scope of practice gradually over the course of five years. Primary care providers have expressed that they appreciated the advantages of having much of the burden alleviated, being able to concentrate on their patient panels and not on the management of the structure.

The CNS for family medicine has proven to be incredibly versatile through this model and able to expand the individual scope of practice through knowledge and experience gained over time. It could be argued, however, that implementing the CNS-led model would increase costs as the CNS costs more than a practice nurse. This is not so. The practice nurse, who already has gained quite a lot of experience working in the individual clinical environment, could be upgraded to the level of a CNS for family medicine, which would cost less than paying for a practice nurse, a clinical manager, and an educator/consultant at the same time.

Though the CNS-led model can be easily implemented in most Commonwealth realms and certainly in all major ones, with the exclusion of Australia, which is currently lagging in terms of nurse prescriber legislation, the CRMC model should be tailored to meet the specifics of the local community, of the staff, and of the patients/clients.

One of the most significant criticisms made against this model by other primary care providers was that not all registered nurses can lead all these multiple roles. While this is a valid argument, the roles are taken on over time to allow the nurse to develop skills and knowledge relative to the particular practice environment. The model does not envisage a revolving door for the CNS. The nurse needs to be committed in the long term to the continued development of both the practice and their roles and skills. The CNS is not a newly qualified nurse. Instead, this professional is a registered nurse with several years of experience, a postgraduate degree, and subsequent practice years, specifically in the family medicine environment. The practice nurse, therefore, seems to be the best candidate. However, the standardization of knowledge and formal training and upgrades are fundamental for the success of the adoption of this role. The CRMC model is designed for scalability. Early-stage discussions with the Government of Alberta and SCPCN focus on replicability and funding strategies for province-wide implementation. By leveraging existing RN Prescriber regulations and structured training, the model is adaptable across multiple jurisdictions.

However, the CRMC model has a limitation that could not be overcome. The CNS-led CRMC model has been tried only in one clinic and has yet to be duplicated anywhere else until now. However, other partner clinics have voiced strong interest in this model, particularly in the formation and hiring of Clinical Nurse Specialists who completed our internships and medical office assistants trained through our program.

### Implications for Nursing Leadership

For the CRMC model to be successful, it must be adapted to the local needs of the individual clinic, even though the core of the model would remain similar in all jurisdictions. This entails opening up to learning from other Commonwealth realms, and this especially goes for Canada, which is often very resistant to anything coming from the outside. This model was created by physicians and nurses who trained in the UK but practiced for many years within the North American framework.

At the same time, for the CRMC model’s success, sharing educational programs has to be free and not-for-profit. Cooperation should be promoted among nurses and regulators between Commonwealth realms instead of all operating within their individual jurisdiction’s bubble. Once a number of clinics adopt this model, an online-based interest group hub should form for clinics to share ideas, continuing professional development programs targeting this model in particular, and to learn from each one another’s experiences.

## 5. Conclusions

The CRMC model offers a fresh approach to managing family medicine clinics by placing a Clinical Nurse Specialist at the centre of operations. This shift has improved teamwork, streamlined patient care, and reduced stress on primary care providers while making clinics more efficient and cost-effective. Although there was some initial hesitation, the model has proven to enhance staff retention, patient access, and overall satisfaction. While it has only been implemented in one clinic so far, the potential for broader adoption is clear. Moving forward, refining training programs and encouraging collaboration across healthcare systems could help bring this model to more clinics, ultimately improving primary care for both providers and patients. The CRMC model presents an innovative, cost-effective solution to primary care challenges in the Commonwealth realms. By leveraging RN Prescribers, clinics can improve accessibility, optimize physician workload, and significantly reduce costs. The collaboration with South Calgary PCN further highlights the model’s potential for broader adoption. Given the strong financial and patient-care benefits, this model serves as a replicable and scalable approach to primary care management in the modern healthcare landscape.

## Data Availability

The data presented in this study are available upon request from the corresponding author.
